# Factors associated with outbreak of diphtheria in Kafanchan, Kaduna State, Nigeria: July-October 2023

**DOI:** 10.11604/pamj.2026.53.118.45929

**Published:** 2026-03-10

**Authors:** Uwaifiokun Julius Okhuarobo, Okpachi Christopher Abbah, Samuel Amifofum Owoicho, Ubong Akpan Okon, Jeremiah Daikwo, Peace David Umar, Isiaq Hadji Shehu, Ibrahim Theophilus Turman, Oyeladun Okunromade, Bola Biliamimu Lawal, Abiodun Egwuenu

**Affiliations:** 1 Nigeria Field Epidemiology and Laboratory Training Programme, Abuja, Nigeria,; 2 Veterinary Public Health and Preventive Medicine, University of Abuja, Abuja, Nigeria,; 3 Nigeria Centre for Disease Control and Prevention, Abuja, Nigeria,; 4 Federal Ministry of Agriculture and Food Security, Abuja, Nigeria,; 5 Public Health Information Surveillance Solutions and Systems, Abuja, Nigeria,; 6 Kaduna State Ministry of Health, Kaduna, Nigeria,; 7 African Field Epidemiology Network, Abuja, Nigeria,; 8 Charite Universitätmedizin, Berlin, Germany

**Keywords:** Diphtheria, vaccination coverage, case-control study, disease outbreaks, risk factors

## Abstract

The year 2023 witnessed the re-emergence of diphtheria in Nigeria. Kafanchan, Kaduna State, reported an unusual surge in cases and deaths. We investigated the outbreak to identify associated risk factors of the disease. We conducted an unmatched (1:2) case-control study. Case-patients were identified through the State linelist and traced to their residence. At the same time, controls were randomly selected from neighbors without symptoms or signs suggestive of diphtheria within the same community. Data was collected using an interviewer-administered structured questionnaire. Bivariate analysis was done to ascertain the odds ratio (OR), while multivariate logistic regression analysis was done to calculate the adjusted odds ratio (aOR). The confidence interval (CI) was set at 95%. A total of 91 case-patients and 182 controls were recruited. The median age of case-patients was 7 years (5-10 years). Forty (44%) of 91 case-patients were females. Sixty-four (70%) of 91 case-patients were from Kafanchan ward B, and the case fatality rate was 23%. Exposure to pentavalent vaccination was found to be associated with protection from diphtheria (aOR: 0.39, 95% CI: 0.20-0.77). Thirty-one (34.07%) of 91 case-patients had contact with someone with respiratory symptoms [OR = 37.2; 95% CI = 16.8-82.6; p <0.001]. Thirty-seven (40.66%) of 91 case-patients had contact with a confirmed case [OR = 25.1; 95% CI = 11.7-53.8; p < 0.001]. Twenty-three (25%) of 91 case-patients had never received the pentavalent vaccine, and the primary reason cited by caregivers was that they were unaware of any benefits of vaccination. The outbreak is attributable to suboptimal pentavalent vaccination coverage. Future outbreaks may be avoided or their impact reduced through improved risk communication and community engagement on the benefits of vaccination, strengthening routine immunization services, early warning surveillance, and prepositioning of diphtheria antitoxin (DAT) and antibiotics.

## Introduction

**Nature of the problem and its public health importance:** diphtheria used to be a prominent contributor to childhood mortality before the advent of vaccines [1,2]. It is a serious bacterial infection caused by toxin-producing *Corynebacterium diphtheriae*. Other toxin-producing strains of Corynebacterium (*C. ulcerans* and *C. pseudotuberculosis*) rarely cause diphtheria. Diphtheria bacteria can spread from person to person, typically via respiratory droplets generated by coughing or sneezing [1,3-5]. In industrialized nations, the incidence of the disease declined significantly in the 1940s following the development of the diphtheria-tetanus-pertussis (DTP) vaccine [1,2]. Similarly, after the World Health Organization's Expanded Programme on Immunization was launched in 1974, the incidence of disease began to decline in less-developed countries [1,2].

Around the world, diphtheria is still endemic in several regions, including the Middle East, Africa, Asia, Eastern Europe, and the Americas (e.g., Haiti and the Dominican Republic) [1,6,7]. The following countries have seen respiratory diphtheria epidemics since 2016: Bangladesh, Burma (Myanmar), Haiti, Indonesia, South Africa, Ukraine, Venezuela, Vietnam, and Yemen [6,7]. Four African countries, Algeria, Guinea, Niger, and Nigeria, have seen a re-emergence of diphtheria outbreaks in 2023. As of October 9, 2023, 14,587 cases had been reported throughout these four countries, with a case fatality rate of 4.1%, and Nigeria alone accounted for more than 90% of the cases [8].

The mainstay of prevention is vaccination. The Nigerian childhood immunization schedule recommends three doses of pentavalent vaccination (Penta-1, Penta-2, and Penta-3) for children in the sixth, tenth, and fourteenth weeks of life, respectively [9]. Achieving high vaccination coverage rates, particularly with the complete series of recommended doses, is essential for establishing herd immunity within the population. The Nigeria Centre for Disease Control and Prevention (NCDC) was notified of suspected diphtheria outbreaks in Kano and Lagos States in December 2022. As of October 2023, over 20 other States had reported outbreaks, including Yobe, Kaduna, Bauchi, and Katsina [10]. Previous diphtheria outbreaks in Nigeria have been documented, with the worst one occurring in 2011 and affecting the rural parts of Borno State in the northeast of the country [10,11].

The index case of diphtheria in Kaduna State was reported to the epidemiology unit of the State Ministry of Health on the 4^th^ of July 2023, in a 4-year-old boy with symptoms of sore throat, cough, fever, and neck swelling. Two weeks later, the State rapid response team was deployed to Kafanchan, Jema'a Local Government Area (LGA) of Kaduna State, to investigate reports of strange deaths among children, which proved to be diphtheria, and the NCDC was notified. Though thirteen other LGAs subsequently reported at least a confirmed case, Kafanchan (in Jema´a LGA) remained the epicenter of the outbreak, accounting for over 70% of confirmed cases across Kaduna State [12].

**Geographic setting:** Kafanchan is the administrative headquarters of Jema´a LGA in the Southern part of Kaduna State, which is in the northwest geopolitical zone of Nigeria. The historic town of Kafanchan is renowned for the railway tracks built in the late 1920s, which connected several cities in Nigeria and were largely responsible for the town's early development [13,14]. Kafanchan is made up of 2 wards (Kafanchan A and Kafanchan B) out of the 12 political wards (Asso, Atuku, Bedde, Gidan-Waya, Godogodo, Jangidi, Kagoma, Kaninkon, Maigizo, and Takau) of the LGA [7,9]. The major ethnic groups include: Tyan, Fantswam, Nikyob, and Mangyang. Kafanchan covers an area of 3,736 km^2^ with an estimated total population of 94,801 (males 50.7%; females 49.3%) [13,15,16]. It has 12 government-owned primary healthcare centers and a general hospital. The under-5 population is 2,416. The main occupation of the people is farming.

**Investigation team and objective:** following the notification by Kaduna State to NCDC, multidisciplinary National Rapid Response Teams were deployed between July and November 2023 to provide support to the State. The support covered several core pillars of the State response, including surveillance and contact tracing; sample management and laboratory; case management, risk communication and community engagement; as well as vaccination. We aimed to investigate the outbreak to identify associated risk factors of the disease in Kafanchan, Kaduna State, Nigeria, from July-October 2023.

## Methods

**Operational case definition:** using the National Technical Guidelines for Integrated Disease Surveillance and Response (IDSR) for Nigeria and other relevant Standard Operating Procedures (SOPs) from NCDC [3,17]. A suspected case was defined as any person in Kaduna State with an illness of the upper respiratory tract characterized by the following: pharyngitis, nasopharyngitis, tonsillitis, or laryngitis AND adherent pseudo-membrane of the pharynx, tonsils, larynx, and/or nose from July 4 - October 31, 2023. A confirmed case was defined as anyone residing in Kaduna State from July 4 - October 31, 2023, with the following: (1) laboratory confirmed: a person with *Corynebacterium spp*. isolated by culture and positive for toxin production, regardless of symptoms; (2) epidemiologically linked: a person who meets the definition of a suspected case and is linked epidemiologically to a laboratory-confirmed case; (3) clinically compatible: a person who meets the definition of a suspected case and lacks both a confirmatory laboratory test result and epidemiologic linkage to a laboratory-confirmed case.

### Case finding methods

**Notification and line-listing:** diphtheria is one of the 25 immediately notifiable case-based priority diseases under the IDSR 3^rd^ edition [17]. Following this guideline, LGA Disease Surveillance and Notification Officers (DSNOs) report cases of diphtheria to the Epidemiology Unit of the State Ministry of Health immediately (or within 24 hours) through the fastest means possible (e.g. phone call, SMS or WhatsApp call/message) and concurrent data capturing using diphtheria case investigation form (CIF) and electronic IDSR into a State linelist and the national Surveillance Outbreak Response Management and Analysis System (SORMAS) [17]. Each diphtheria case report was examined and cross-referenced with the State linelist and SORMAS database to identify individuals who met the given case definition.

**Active case search:** per the IDSR Technical Guideline [17], on the steps in conducting an active case search, health facilities and communities were visited in search of additional cases. A thorough search was conducted in health facilities/communities where cases have been reported, retrospectively reviewing outpatient and inpatient registers from July 2023 to October 2023. The aim was to identify any missed/additional suspected cases and deaths. Close attention was paid to other patients who might have similar signs and symptoms in keeping with diphtheria. The team also asked health workers to check for similar cases in neighboring health facilities/communities and where they may have visited.

A community active case search was carried out using a house-to-house case search approach, where the team visited every house in the community with a designated community guide. Contacts of confirmed cases were also searched, traced, and monitored for 10 days based on the incubation period of diphtheria using recommended contact listing and monitoring forms from the IDSR technical guideline [3,17]. All missed or suspected cases of diphtheria discovered from the active case search, from both health facilities and communities, as well as during contact tracing and monitoring, were entered into an adapted linelist from the IDSR technical guideline for further investigation. Based on available data collected from each individual, they were classified based on case definitions of a confirmed case (Epidemiologically linked, clinically compatible or laboratory-confirmed).

**Verbal autopsy:** during active case search, a verbal autopsy was conducted to get anamnestic data from family members or guardian/caregivers of persons who died after developing symptoms of diphtheria but were not reported to health authorities or captured by the surveillance system [18]. Any death qualified if it met the following set definition: “any death of a family member who, between July 4, 2023 and October 31, 2023, resided in Kafanchan, Jema´a LGA, Kaduna State, Nigeria; and who within 10 days before death had some or all of the following: an illness of the upper respiratory tract characterized by pharyngitis, nasopharyngitis, tonsillitis or laryngitis and adherent pseudo-membrane of the pharynx, tonsils, larynx and/or nose.” Any identified death was cross-referenced with the State linelist to avoid duplication.

### Analytical study design and rationale

**Case-control study:** an unmatched case-control study was conducted in the Kafanchan community between October 7, 2023, and October 31, 2023. Most reported cases of diphtheria in Kaduna State were from Kafanchan [12]. A case-patient was defined as any confirmed (epidemiologically linked, clinically compatible, or laboratory confirmed) case from Kafanchan from July 4, 2023, to October 31, 2023. For each case-patient, 2 neighborhood controls were selected. Controls were defined as those without respiratory illness or diphtheria during the same period. Controls were chosen through a random selection process initiated by spinning a pen on the ground in front of the case-patient's residence. Interviewers followed the direction indicated by the spun pen to reach the nearest neighborhood household. In cases where a household had multiple individuals, balloting was done to pick the controls. If no suitable control candidate was found, the interviewer moved on to the next household until two control subjects per case-patient were successfully enrolled in the study [19,20].

**Data management and analysis:** data were collected on different domains based on socio-demographic characteristics, predisposing risk factors, as well as vaccination status/history from parents/caregivers of both case-patients and controls using an interviewer-administered structured questionnaire. This was done after obtaining informed consent from the participants´ parents/caregivers. Interviewers were blinded to the research hypothesis to avoid researcher bias. Where a participant was above the recommended legal age of 18 years old, he/she provided the consent needed to administer the questionnaire. The questionnaire was adapted from the World Health Organization´s Diphtheria Toolbox Surveillance Standards manual on Diphtheria Vaccine Preventable Disease [21] and a similar study carried out in the Lao People´s Democratic Republic [19]. The questionnaire was adapted using the Kobo Toolbox, and data collection was aided by the Kobo Collect v2023.1.2 on an Android device [22]. Pre-testing of the questionnaire was done in Takau ward of Jema´a LGA, Kaduna State, Nigeria, on October 7, 2023, while interviews, using an interviewer-administered structured questionnaire, were conducted in Kafanchan (Kafanchan A and B wards), Jema´a LGA, Kaduna State, Nigeria, from October 8, 2023, to October 31, 2023.

Data collected on the Kobo Toolbox platform was downloaded in Excel format (Microsoft Excel 365) [23]. Epi Info version 7.2.4.0 [24] was utilized for analysis of the data. Descriptive statistics were utilized in summarizing the data. Bivariate analysis was performed to ascertain the odds ratio (OR) of associations between independent variables and confirmed diphtheria case-patients. While a multivariate logistic regression analysis was done to calculate the adjusted odds ratio (aOR). The Confidence Interval (CI) was set at 95%. The significance level was set at 5% while the statistical test was done using Chi-square.

**Laboratory methods:** nasal and pharyngeal swabs were collected by trained officers, well-packaged, and transported the samples through designated NCDC-assigned courier services to the National Reference Laboratory in Abuja for culture. All confirmed cases were referred to the nearest isolation center for treatment.

**Reactive mass vaccination campaign:** with support from the National Primary Health Care Development Agency (NPHCDA), reactive mass vaccination with pentavalent and tetanus diphtheria (Td) vaccines was administered to the targeted population in Jema´a LGA and other affected LGAs in Kaduna State. Children from 6 weeks to less than 4 years were given the pentavalent vaccine, while those from 4 years to 14 years were given the tetanus diphtheria (Td) vaccine.

**Ethical consideration:** all data utilized in this study were gathered as part of the 2023 diphtheria outbreak investigation and response in Kafanchan, as well as the entire Kaduna State, Nigeria. The Kaduna State Ministry of Health´s ethics committee approved this study. Approval codes include MOH/ADM/744/VOL.1/111044 and NHREC/17/03/2018. Consent from a parent or legal guardian was sought for all children who were less than 18 years of age. Confidentiality was upheld in every part of the study, and permission was obtained from community leaders in every community the team visited before commencement of any field work.

## Results

**Figure 1:**
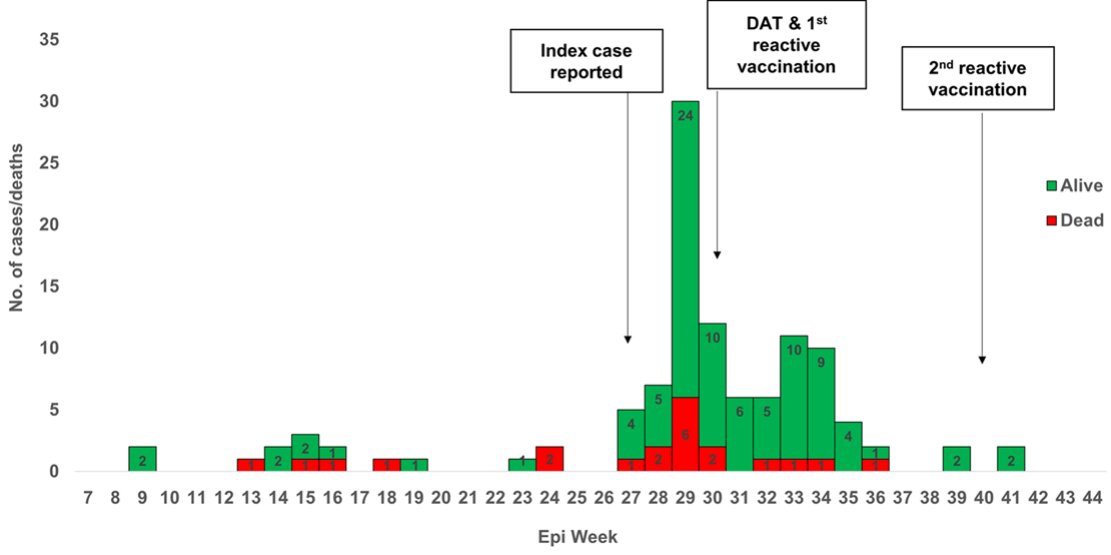
distribution of case-patients and deaths by Epi Week in Kafanchan, Kaduna State, July-October 2023

A total of 91 case-patients and 182 controls were recruited. The median age of case-patients was 7 years (interquartile range: 5-10 years) and controls 5 years (interquartile range: 4-8 years). Thirty-two (32) (35%) of 91 case-patients were less than 5 years, while the 6-10 age group accounted for 42 (46%) of 91 case-patients (Table 1). Forty (44%) of 91 case-patients and 80 (44%) of 182 controls were females, respectively. Sixty-four (70%) of 91 case-patients were from Kafanchan ward B, and 21 (23%) of 91 case-patients were dead. Hence, the case fatality rate (CFR) of the outbreak was 23%. A distribution of case-patients & deaths by Epidemiological Week (Epi Week) is shown in Figure 1.

**Table 1:** socio-demographic characteristics of respondents in Kafanchan, Kaduna State, Nigeria, (n = 273)

Variables	Frequency			Total (%)
	n	Case; (n) (%)	Control; (n) (%)	
**Age group (years)**				
< 6	125	32 (25.60)	93 (74.40)	46
6 - 10	113	42 (37.17)	71 (62.83)	41
11 - 15	30	12 (40.00)	18 (60.00)	11
>15	5	5 (100.00)	0 (0.00)	2
**Sex**				
Male	153	51 (33.33)	102 (66.67)	56
Female	120	40 (33.33)	80 (66.67)	44
**LOE of the respondent**				
None	30	7 (23.33)	23 (76.67)	11
Nursery	96	27 (28.13)	69 (71.88)	35
Primary	120	39 (32.50)	81 (67.50)	44
Secondary	26	17 (65.38)	9 (34.62)	10
Tertiary	1	1 (100.00)	0 (0.00)	0
**LOE of mother**				
None	9	5 (55.56)	4 (44.44)	3
Nursery	9	3 (33.33)	6 (66.67)	3
Primary	65	20 (30.77)	45 (69.23)	24
Secondary	173	57 (32.95)	116 (67.05)	63
Tertiary	17	6 (35.29)	11 (64.71)	6
**LOE of father**				
None	3	1 (33.33)	2 (66.67)	1
Nursery	3	1 (33.33)	2 (66.67)	1
Primary	17	6 (35.29)	11 (64.71)	6
Secondary	190	66 (34.74)	124 (65.26)	70
Tertiary	60	17 (28.33)	43 (71.67)	22
**Mother’s occupation**				
Farmer	2	2 (100.00)	0 (0.00)	1
Trader	67	24 (35.82)	43 (64.18)	25
Housewife	178	57 (32.02)	121 (67.98)	65
Others	26	8 (31.00)	18 (69.00)	9
**Father’s occupation**				
Farmer	24	4 (17.00)	20 (83.00)	9
Trader	161	54 (34.00)	107 (66.00)	59
Civil Servant	15	7 (46.67)	8 (53.33)	5
Artisan	3	1 (33.3)	2 (66.67)	1
Clergy	2	0 (0.00)	2 (100.0)	1
Health worker	2	1 (50.00)	1 (50.00)	1
Others	66	24 (36.36)	42 (63.64)	24

LOE: Level of education

Thirty-one (34.07%) of 91 case-patients and 173 (95.1%) of 182 controls had contact with someone with respiratory symptoms [OR = 37.2; 95% CI = 16.8-82.6; p <0.001] within 10 days before onset of symptoms. Thirty-seven (40.66%) of 91 case-patients and 172 (94.5%) of 182 controls had contact with someone confirmed to have diphtheria [OR = 25.1; 95% CI = 11.7-53.8; p < 0.001] within 10 days before onset of symptoms. Eighteen (19.8%) of 91 case-patients and 36 (19.8%) of 182 controls had contact or consumed unpasteurized dairy products within 10 days before onset of symptoms [OR = 1.00; 95% CI = 0.53-1.88; p = 1,00]. Seventeen (18.7%) of 91 cases and 30 (16.5%) of 182 controls had contact with domesticated animals such as cats, dogs, and pigs within 10 days before onset of symptoms [OR = 0.86; 95% ci = 0.45-1.66; p = 0.78] (Table 2).

**Table 2:** risk factors associated with diphtheria outbreak in Kafanchan, Kaduna State, Nigeria, July-October 2023, (n = 273)

Risk factors	Cases (%); (n=91)	Control (%) (n=182)	Odd ratio	95% confidence interval	P-value
**Contact with anyone who had respiratory symptoms 10 days before the onset of symptoms**					
Yes	31 (34.07)	173 (95.05)	37.20	16.75-82.64	< 0.001
No	60 (65.93)	9 (4.95)			
**Contact with anyone who was confirmed to have diphtheria within 10 days before the onset of symptoms**					
Yes	37 (40.66)	172 (94.51)	25.10	11.71-53.81	< 0.001
No	54 (59.34)	10 (5.49)			
**Travel history within 10 days before the onset of symptoms**					
No	91 (100.00)	182(100.00)	NA	NA	NA
**Contact with someone who travelled within 10 days before the onset of symptoms**					
No	91 (100.00)	182(100.00)	NA	NA	NA
**Contact/consumption of unpasteurized dairy products within 10 days before the onset of symptoms**					
Yes	18 (19.78)	36 (19.78)	1.00	0.53-1.88	1.000
No	73 (80.22)	146(80.22)			
**Contact with domesticated animals such as cats, dogs, and pigs within 10 days before the onset of symptoms**					
Yes	17 (18.68)	30 (16.48)	0.86	0.45-1.66	0.780
No	74 (81.32)	152(83.52)			
**Pentavalent vaccine 1**					
Yes	68 (74.73)	160(87.91)	0.41	0.21-0.77	0.0056
No	23(25.27)	22(12.09)			
**Pentavalent vaccine 2**					
Yes	53(58.24)	115(63.19)	0.81	0.49-1.36	0.430
No	38(41.76)	67(36.81)			
**Pentavalent vaccine 3**					
Yes	38(41.76)	77(42.31)	0.98	0.59-1.63	0.930
No	53(58.24)	105(57.69)			
**Has the respondent ever received the tetanus-diphtheria vaccine?**					
Yes	64(70.33)	146(80.22)	0.58	0.33-1.04	0.067
No	27(29.67)	36(19.78)			
**Does the respondent have a vaccination card?**					
Yes	60(65.93)	140(76.92)	0.58	0.33-1.01	0.053
No	31(34.07)	42(23.08)			

NA = Not applicable

Of the 273 respondents for this study, 267 (98%) were sure of ever receiving any dose of pentavalent vaccine or not; out of which only 230 (86%) had received at least one dose of pentavalent vaccine, while the remaining 37 (14%) never received any dose. Thirty-eight (42%) of 91 case-patients had received the 3rd dose of the pentavalent vaccine, [OR: 0.98, CI: 0.59-1.63]. Twenty-three (25%) of 91 case-patients had never received the pentavalent vaccine (zero dose) [OR: 0.41, CI: 0.21-0.77] (Table 2), and the major reason given by caregivers was not being aware of any benefit of the vaccination. Exposure to pentavalent vaccination was found to be associated with protection from diphtheria infection (aOR=0.39, 95% CI=0.20-0.77) (Table 3).

**Table 3:** logistic regression analysis for diphtheria vaccines taken

Covariates	Adjusted odds ratio	95% CI		Coefficient	SE	Z-statistic	P-value
Has the respondent ever received the pentavalent vaccine? (Yes/no)	0.3927	0.2004	0.7697	-0.9346	0.3433	-2.7226	0.0065
Has the respondent ever received Td vaccine? (Yes/no)	0.6646	0.3660	1.2068	-0.4085	0.3043	-1.3424	0.1795

CI: confidence intervals; SE: standard error; Td: tetanus diphtheria

## Discussion

This study has shown the importance of pentavalent vaccination in limiting the incidence of diphtheria infection. This implies that children who received the vaccine had protection from the infection. It therefore means that suboptimal pentavalent vaccination coverage results in vulnerability of the population to the infection and attendant outbreaks [25,26] as in the case of Kafanchan. In this study, the age groups most affected (0 - 5 years and 6 - 10 years) were similar to those reported in previous outbreaks [2,27,28], indicating that diphtheria is a childhood disease. It is important to stress that children are particularly susceptible to contracting diphtheria because of their developing immune systems and susceptibility to infections. The implication of this, therefore, shows possible gaps in the effectiveness of the available disease prevention measures and vaccination strategies [1,2]. The 2021 National Immunization Coverage Survey (NICS) report showed that Kaduna State had 60% coverage of the 3^rd^ dose of pentavalent vaccine, which is below the acceptable target of 90% [29]. This may have contributed to lowering herd immunity in the population.

Most parents of case-patients had a secondary level of education. Parents with this level of education may not have been educated enough to make informed decisions about improving vaccine uptake for their children. The NICS report, however, showed that increasing levels of education among mothers improved vaccine uptake [29]. The CFR in this outbreak is 23%, which is higher than reported mortality in the literature of 5 to 10% and comparable to reported figures as high as 40%, especially in areas with poor vaccination coverage [10]. This alarming CFR may be a reflection of systemic gaps in outbreak preparedness and case management. Late detection of the outbreak, as seen from the epidemic curve (Figure 1) and the late administration of diphtheria antitoxin (DAT), which has been shown to reduce the incidence of complications and mortality [30], contributed to poor outcomes. These results emphasize how crucial it is to strengthen early warning for the diphtheria surveillance system and prepositioning of DAT and antibiotics to promptly detect, respond, and manage cases during outbreaks and to reduce the CFR as much as possible.

The result of the study showed little or no sex predilection towards diphtheria infection. The majority of the case-patients and deaths were from Kafanchan B ward. The reason behind this is unclear. It may be because of poorer herd immunity among children in that ward, which is usually associated with a high burden of diphtheria [31]. The suboptimal coverage of the 3rd dose of the pentavalent vaccine in Kaduna State, as reported in the 2021 NICS [29], may explain the high burden of infection in this community.

The results of this study showed that the major mode of transmission is through human-to-human contact, particularly through contact with symptomatic individuals or confirmed cases [1,5]. There may be interesting new information about possible risk factors for diphtheria, especially in relation to contact or consumption of unpasteurized dairy products and contact with domesticated animals like dogs, cats, and pigs [32,33]. Although these factors did not significantly increase the risk of contracting the disease in the current study, their relationship to the predominant cattle rearing industry in northern Nigeria is worth investigating further. Unpasteurized dairy products have been linked to the spread of several infectious diseases, including diphtheria [34,35]. Hence, this study provides an opportunity for future research on the value of an integrated “One Health” surveillance system and the gaps in understanding the need to explore the potential for environmental and animal reservoirs and the accompanying transmission dynamics.

The study findings reveal a notable observation regarding the proportion of individuals who have not received any dose of the pentavalent vaccine compared to those who have completed the recommended three doses. While the proportion of zero doses may appear low in comparison to fully vaccinated individuals, it remains significant in the context of assessing overall herd immunity against diphtheria. This aligns with the NICS report, which showed below-average vaccination rates for Kaduna State [29]. It is therefore imperative to improve pentavalent vaccination coverage to avoid disease outbreaks. This study also found that some caregivers were ignorant of the benefits of vaccinating their children, which is also in keeping with findings from the NICS report that revealed that lack of knowledge or information about vaccination was the major reason given why children were not fully vaccinated [29]. This raises concerns about the effectiveness of risk communication and community engagement strategies for immunization services.

There was a significant association between exposure to the pentavalent vaccine and protection against diphtheria in this study. This emphasizes how crucial pentavalent vaccination is in granting immunity against this potentially fatal infectious illness. When given per the recommended schedule and dosage, the pentavalent vaccine exposure minimizes an individual's susceptibility to diphtheria and confers immunity against the illness [1-5]. By prioritizing vaccination efforts and strengthening immunization programs, public health authorities can mitigate the burden of diphtheria and prevent outbreaks of the disease in susceptible populations.

**Limitations:** there could be a difference in recollection between cases and controls if participants don't recall past exposures or immunization histories. Secondly, the unique context of Kafanchan, Kaduna State, may make study findings inapplicable to other contexts or populations. Additional limitations include a small sample size for some risk factors and potential residual confounding.

## Conclusion

The strong correlation between exposure to the pentavalent vaccine and protection against diphtheria was a significant finding of this study. Pentavalent vaccination is crucial in limiting the transmission and occurrence of the disease. This study emphasizes how important it is to maintain high vaccination rates and to ensure that everyone in the community has access to immunization services. Future diphtheria outbreaks can be prevented by narrowing vaccination coverage gaps through catch-up vaccination campaigns and integration of risk communication and community engagement during routine immunization. Apart from vaccination, reducing the mortality from diphtheria outbreaks requires adequate investment in health system preparedness, including enhanced surveillance, stockpiling of DAT, routine sensitization of clinicians, and improved coordination between the surveillance system and treatment centers.
